# The influence of kinematic conditions and design on the wear of patella-femoral replacements

**DOI:** 10.1177/0954411913518910

**Published:** 2014-02

**Authors:** Raman Maiti, John Fisher, Liam Rowley, Louise M Jennings

**Affiliations:** 1 Institute of Medical and Biological Engineering, School of Mechanical Engineering, University of Leeds, Leeds, UK; 2 DePuy Synthes Joint Reconstruction, A Johnson & Johnson Company, Warsaw, IN, USA

**Keywords:** Knee replacement, patella-femoral joint, wear, arthroplasty, in vitro

## Abstract

The success rate of patella-femoral arthroplasty varies between 44% and 90% in 17 years of follow-up. Several studies have been performed previously for assessing the surface wear in the patella-femoral joint. However, they have not included all six degrees of freedom. The aim of this study was to develop a six-axis patella-femoral joint simulator to assess the wear rate for two patellae designs (round and oval dome) at different kinematic conditions. An increase in patellar rotation from 1° to 4° led to a significantly (*p*<0.049) increased wear rate of round dome from 8.6 mm^3^/million cycles to 12.3 mm^3^/million cycles. The wear rate for oval dome increased from 6.3 mm^3^/million cycles to 14.5 mm^3^/million cycles. However, the increase was nonsignificant (*p*>0.08). The increase in wear rate was likely due to the higher cross shear. A decrease in patellar medial lateral displacement from passive to constrained resulted in a nonsignificant reduction in wear (*p*>0.06). There was no significant difference in wear rate between the two patellae designs (*p*>0.28). The volumetric wear under all conditions was positively correlated with the level of passive patellar tilt (rho>0.8). This is the first report of preclinical wear simulation of patella-femoral joint in a six-axis simulator under different kinematic conditions.

## Introduction

More than 2.7 million joint replacement surgeries have been performed globally.^1^ Among them, 1.3 million are total knee joint replacements.^1,2^ To date, the question of whether to replace the patella or not remains uncertain.^3–7^ The popularity in replacing the patella during total knee replacements varies geographically with the following countries: the United States (90%), Denmark (76%), Australia (43%), England and Wales (33%), Sweden (14%) and Norway (11%).^8–11^

The success rate of patella-femoral arthroplasty lies between 44% and 90% in 17 years of follow-up studies.^8,12^ The major reasons for the failures in patella-femoral joint replacements are loosening, infection, fracture, instability, maltracking, wear and overstuffing.^13–15^ It has been widely reported that failures in artificial tibia femoral joint were due to wear debris–induced osteolysis leading to implant loosening.^16,17^ Ellison et al.^18^ reported a 19% increase in the generation of wear debris when patella-femoral joint (PFJ) particles were included alongside the tibia femoral joint in an in vitro wear simulator comprising five degrees of motion. The generation of wear debris is dependent on many factors, including the surface roughness of the metallic femoral component, artificial knee joint design, oxidative degradation of polyethylene, patient activities, surgical alignment and kinematic input profiles.^19,20^

Wear of the artificial PFJ has been investigated by several authors. The conditions of testing were, however, limited to a maximum five degrees of freedom or less. Two degrees of freedom (flexion extension (FE) and anterior posterior (AP) load) were used for wear assessment by Hsu and Walker^21^ and Burroughs et al.^22^ Korduba et al.^23^ used controlled and uncontrolled medial lateral displacement (also referred to as patellar shift) in addition to flexion extension and patellar compressive force (also referred to as axial load) for load and kinematic inputs. The method of wear assessment was gravimetric measurement by Korduba et al.^23^ The other authors mentioned above, Hsu and Walker^21^ and Burroughs et al.,^22^ used Fuji films and grading methods for quantifying the wear. The method adopted was by comparison based on visual inspection only.

An early study by Hsu and Walker^21^ studied all plastic- and metal-backed polyethylene patella buttons cemented to a rigid foam polyurethane backing under constant loads of 750–1500 N and FE of 55° to 100° for 5000 cycles at a cycle rate of 32 Hz. The cycle rate and constant load did not represent the physiological scenario. The lubrication was provided by a sponge soaked in distilled water. The metallic-based polyethylene was damaged by the femoral part penetrating through the plastic in the patella buttons. Burroughs et al.^22^ investigated the wear of conventional and highly cross-linked ultrahigh molecular weight polyethylene (UHMWPE) patella buttons at 2450 N dynamic AP load and 10° to 70° FE rotation in a six-station knee simulator (MTS, Minnesota, USA). The conventional polyethylene was GUR 1020, which had been sterilised in oxygenless nitrogen environment and aged in air for 35 days. The highly cross-linked polyethylene was GUR 1050, sterilised using ethylene oxide gas. Cracks and delamination were found in the conventional polyethylene due to the 35-day ageing process. However, the highly cross-linked polyethylene patella buttons reported no such failures. Korduba et al.^23^ reported wear of conventional polyethylene in the range of 2.78–5.0 mm^3^/million cycles (MC) at conditions similar to Burroughs et al.,^22^ with the addition of controlled and uncontrolled medial lateral (ML) displacement.

All the studies reported so far have used two or three degrees of motion, FE rotation and AP load in their investigation, which replicated a very limited representation of the in vivo scenario. In addition, the wear was assessed qualitatively using visual inspection with the exception of Korduba et al.^23^ Authors who have considered the effect of additional patellar motions are Ellison et al.^24^ and Vanbiervliet et al.^25^ Ellison et al.^24^ considered four controlled and two passive degrees of freedom for their investigation on PFJ wear. However, the patellar ML displacement was limited to 1 mm. The wear was analysed under the action of axial load (200–1200 N), abduction adduction (AA) rotation (also referred to as patellar rotation) (0°–1°), FE (0–22°) and superior inferior (SI) displacement (−5 to 20 mm).^24^ Compared to Ellison et al.,^24^ Vanbiervliet et al.^25^ used lower compressive force (−50 to 400 N), high patellar rotation (−10° to 10°), higher FE (0° to 40°), and the ML and SI displacements each varied from −10 to +10 mm. The sixth degree of freedom, internal external (IE) rotation (also referred to as patellar tilt), was constrained at 0° and 4° compared to passive patellar tilt movements in Ellison et al.^24^ Dome-shaped patellae were used for both investigations. The wear rates in both studies were determined using gravimetric measurements and they varied from 0.34 mm^3^/MC^23^ to 3.13 mm^3^/MC.^24^

The wear rate in the patella-femoral joint was found to be much lower than the wear rate in the tibia femoral joint (5–40 mm^3^/MC).^19,26–28^ However, a retrieval study of patella replacements has shown a high variation of wear rate (1.3–45.16 mm^3^/year)^29^ compared to in vitro investigations.^24^ The retrievals study, however, included implants sterilised in air leading to oxidation and delamination fatigue failures. Also, it can be difficult to compare with simulation studies as often many simulations use an average activity profile in an average patient where the implant has been perfectly ‘surgically’ aligned. Hence, the parameters in vivo are not limited to in vitro conditions. To this date, the effect of six degrees of freedom on the wear of modern UHMWPE buttons with high and normal physiological conditions has not been investigated.

The aim of this study was to develop a new method for determining wear of the PFJ and to evaluate the effect of kinematics on wear and, specifically, to determine the influence of kinematics and patella button designs on the wear of the artificial patella-femoral joint using a new six-axis PFJ simulator.

## Materials and methods

The commercially available Press Fit Condyle (PFC) Sigma design was used in this study (DePuy Synthes Joint Reconstruction, Warsaw, Inc, USA). The design consisted of metallic Co-Cr-Mo femoral component (right knee size three) and UHMWPE 1020 gamma vacuum foil (GVF) patella buttons (round and oval dome). Five sets of each design were tested.

**Figure 1.**
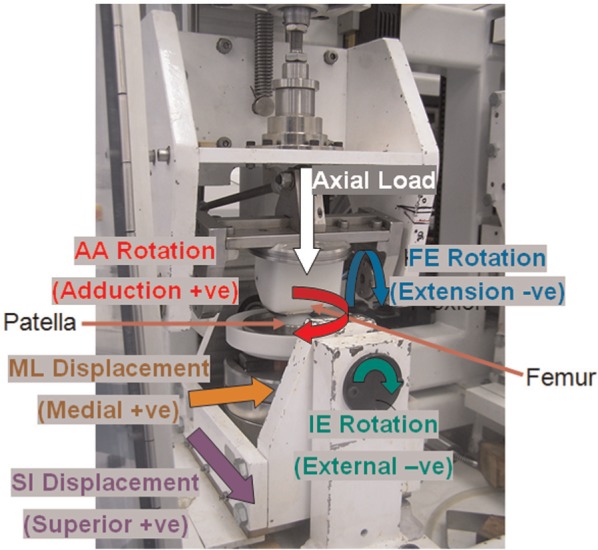
Schematic diagram of patella-femoral joint showing six degrees of freedom and polarities of each degree in simulator.

**Figure 2.**
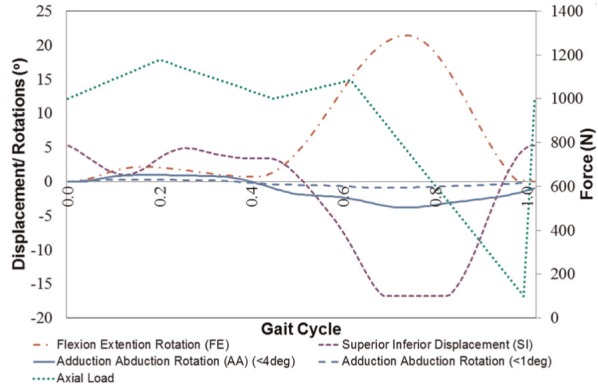
Input kinematics of the wear test.^24^

The Leeds Prosim six-station knee simulator (Simulation Solutions Ltd, Stockport, UK) is a platform for wear testing at controlled loading and kinematic conditions. It has been used for over a billion cycles of knee wear testing.^19,20,26,30–33^ The simulator was modified to create a patella-femoral joint simulator by increasing the tibial cradle length. A linear bearing was introduced in the fixture to obtain the sixth degree of freedom, that is, ML displacement. Each station in the simulator permitted six degrees of freedom, of which four (FE, Axial load, SI and AA rotation) were controlled as shown in Figures 1 and 2.

Four degrees of freedom were controlled and the other two, ML displacement and patellar tilt, were not constrained. ML displacement and patellar tilt were dependent on the geometry of the articular surface and were, hence, left unconstrained. The active motions (FE, SI, AA, axial load) were originally gathered from the combination of in vivo data from natural and artificial joints as the data from artificial joint alone were not sufficient for creating the control strategy.^24^ The FE rotation was based on an artificial joint data^34^ with a maximum flexion angle of 22° (Figure 2). As the data on artificial joints were not based on the same coordinate system, the global coordinate system of a healthy natural joint^35^ was used. Patellar SI motion was based on artificial joint kinematics by Halloran et al.^34^ and varied from 5 to −17 mm. As data for AA rotation were not presented in the literature for an artificial joint, healthy joint patellar rotation was selected^35^ and scaled to post-total knee replacements (TKR) data reported from Halloran et al.^34^ The maximum AA rotation was either 1° or 4° depending on the test conditions. The AA rotation increases cross shear, a significant variable in the wear of polyethylene, which was the rationale behind selecting two different patellar rotations.^36^ The axial load was based on the assumption of 75 kg body weight with maximum axial force of 1177 N. The trend on the axial force was based on the data from Lafortune et al.,^37^ Zavatsky et al.,^38^ Van Eijden et al.^39^ and Nordin,^40^ justifying the evidence that PFJ force is proportional to the tibial flexion angles for angles less than 60°. The test was conducted under three kinematic conditions as shown in Table 1. Low AA rotation (<1°) with passive ML displacement, described as ‘Intermediate’, was the most physiologically relevant condition for an ‘Average’ patient according to the available literature.^24^ High AA rotation (<4°) with passive ML displacement, described as ‘High’, was used to investigate the influence of rotation on wear, and low AA rotation with constrained ML displacement, described as ‘Low’, was used to investigate the influence of ML displacement.

**Table 1.** Conditions and test duration for the wear test.

Conditions	Design type	Number of million cycles
High patellar rotation (<4°) and passive ML displacement (‘High’)	Round	6
	Oval	3
Low patellar rotation (<1°) and passive ML displacement (‘Intermediate’)	Round	3
	Oval	3
Low patellar rotation and constrained ML displacement (<1.6 mm) (‘Low’)	Round	3
	Oval	3

Newborn calf serum of 25% volume (Seralab, Haywards Heath, West Sussex, UK) supplemented with 0.03% of sodium azide solution was the lubricant used for all the tests. Sodium azide was used to minimise the bacterial growth. The serum mixture was changed every 330,000 cycles.

Two specimens were used as soak controls, minimising the error arising from the absorption of fluid in polymers as outlined in tibia femoral wear testing in ISO 14243-2^41^ when measuring gravimetrically. The specimens were measured before and after every MC using a Mettler AT201 digital balance (Mettler Toledo Inc., Columbus, Ohio, USA) with a readability of 0.01 mg, and the wear volume was calculated with ±95% confidence limit. Two-dimensional contacting profilometry (Form Talysurf series, Taylor Hobson, UK) was used to measure the average surface roughness R_a_ of the wear area in the articulating components before and after the wear test and presented in mean ±95% confidence limit. To determine the wear area, the boundaries of the wear scar were marked with nonpermanent ink and captured in ‘JPEG’ format using a camera (Cannon SLR 80). The wear area was then calculated using Image ProPlus (MediaCybernetics, MD, USA).

The patellar tilt was measured three times every 300,000 cycles using an oscilloscope connected to a potentiometer. The tilt was compared with the wear volume corresponding to the same station to derive a relation between the two parameters. Statistical analysis using one-way analysis of variance (ANOVA) (significance taken at *p*<0.05) and post hoc Fisher’s least square difference analysis were performed in IBM SPSS statistics software (IBM Hampshire, UK) to investigate the significance between and within groups. Spearman correlation analysis (rho) was used to establish a correlation between volumetric wear and tilt and presented with *p* value.

## Results

**Figure 3.**
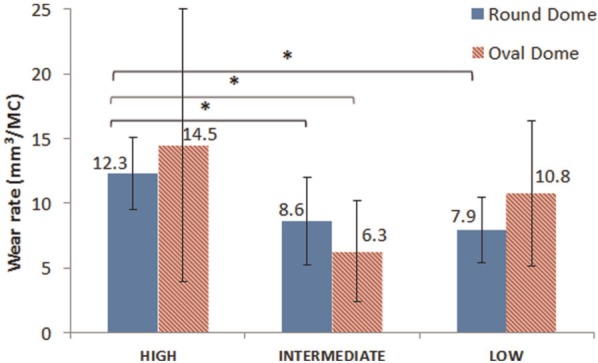
Wear rate with 95% confidence limit under the three test conditions (*n* = 5).

The wear rates for both patellae button designs were highest at ‘high’ kinematic conditions, as shown in Figure 3. At this condition, the wear rates of round and oval dome patellae buttons were 12.3 ± 2.8 mm^3^/MC and 14.5 ± 10.5 mm^3^/MC, respectively. With a decrease in rotation from 4° to 1° and similar ML displacement, the wear rate of round dome significantly decreased to 8.6 ± 3.4 mm^3^/MC (*p*<0.049). The wear rate of the oval dome patella also decreased to 6.3 ± 3.9 mm^3^/MC, but this was not significant (*p*>0.08) compared to the wear rate of oval dome at higher kinematics. With a further decrease in ML displacement from passive (3.5 mm) to constrained (1.6 mm) and similar rotation (<1°), the wear rate was 7.9 ± 2.5 mm^3^/MC and 10.8 ± 5.9 mm^3^/MC for round and oval dome patellae buttons, respectively. However, the wear rate was not significantly different compared to the wear rate at the passive ML displacement condition (*p*>0.06). There were no significant differences in the wear rates between the two designs of patellae buttons for all three kinematic conditions (*p*>0.28). In addition, post hoc analysis using Fisher’s least square difference showed no outliers within the groups.

**Figure 4.**
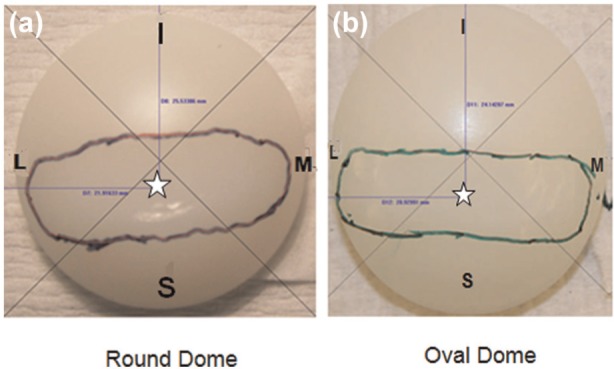
Shape and centroid position (star) of wear scar in patellae buttons. The position of the patella is labelled M: medial, L: lateral, S: superior and I: inferior – (a) round dome and (b) oval dome.

The overall wear scar area for the round dome and oval dome patellae at the end of the test was 39.9% and 40.5% of total articulating surface area, respectively (Figure 4). The wear scar was ‘bow tie’ shape and located in the superior bottom half of the patella buttons. The centroid of the wear scar, for both the dome patellae buttons, was located in the bottom superior and distributed along medial lateral quadrants.

The average surface roughness (R_a_) for the round dome patella button increased significantly (*p*>0.20) from 1.1 ± 0.23 µm to 2.9 ± 1.72 µm, and for the oval dome patella button, the R_a_ increased significantly 0.8 ± 0.07 µm to 2.01 ± 1.5 µm (*p*<0.04). The average roughness for the femur in contact with round and oval dome patellae buttons significantly increased from 0.03 ± 0.01 µm to 0.07 ± 0.02 µm in both cases (*p*<0.01).

**Figure 5.**
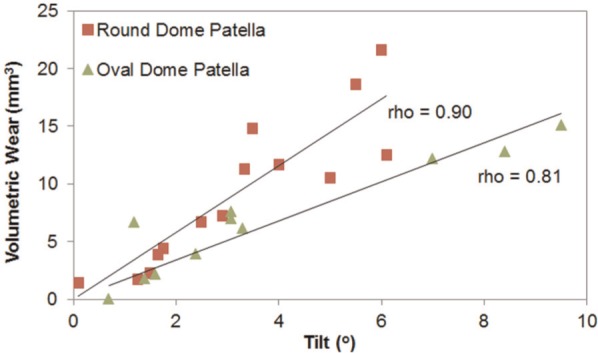
Relation of volume loss with tilt using Spearman correlation coefficient (rho).

As shown in Figure 5, the volumetric wear increased with increasing patellar tilt. The Spearman correlation (rho) was 0.90 for round dome and 0.81 for oval dome patellae buttons, respectively. Also, there was significant difference in the correlation between the tilt and wear volume of the two dome patellae (*p*<0.01).

## Discussion

A six-axis PFJ wear simulator has been successfully created by modifying the Leeds/Prosim knee simulator. Wear of the PFJ joint has been successfully quantified for two patellae designs at three kinematic conditions for a total of 21 MC: 12 MC for round dome and 9 MC for oval dome patellae buttons. In this project, level gait analysis has been investigated, which is the first step in the development of any joint simulations.

The wear rate decreased from 12.3 ± 2.8 mm^3^/MC to 8.6 ± 3.4 mm^3^/MC for round dome and 14.5 ± 10.5 mm^3^/MC to 6.3 ± 3.9 mm^3^/MC for oval dome, respectively, with decrease in AA rotation from 4° to 1°. Unidirectional sliding of UHMWPE is highly resistant to wear due to the alignment of the polymer in that direction causing strain hardening. As the motion gets multidirectional, with the addition of rotation or displacement in the perpendicular direction, strain softening occurs in this direction, thereby increasing wear. This phenomenon is termed as cross shear, which is one of the major causes of higher wear rate in polymers.^19,20,42^ Therefore, the fourfold decrease in AA rotation caused a decrease in cross shear, which led to the lower wear rate.

A decrease in the ML displacement caused no significant change in the wear rate of the patellae buttons. The difference in the actual displacement between the two conditions was very small (2 mm), and hence may not have been large enough to result in a significant change in the wear rate. The patella button articulated 3.5 mm medially to follow the femoral groove. The high 95% confidence limits of the wear rates may have been due to the large difference in the tilt between stations. Post hoc analysis of the wear rate within each data group showed no outliers.

The wear rates of the oval and round dome patellae buttons at low AA rotation and ML displacement were found to be higher than the wear rate found by Ellison et al. (3.1 ± 1.7 mm^3^/MC),^24^ Vanbiervliet et al.^25^ (0.3–0.9 mm^3^/MC) and Korduba et al.^23^ (2.8–5.0 mm^3^/MC). The lower wear rate in the study by Ellison et al.^24^ was possibly due to different experimental setup conditions and lower ML displacement as compared to this study. The difference in experimental set up and kinematic input conditions of total knee replacements has been shown to have a significant influence on the wear.^43^ Furthermore, the variation of patellar tilt from Ellison et al.^24^ was not reported and hence, could not be compared with the current study which could be an important factor.

Vanbiervliet et al.^25^ predicted a patella wear rate 0.91 ± 0.21 mm^3^/MC, which was 35 times lower than the wear rate in this study. The axial load in their study was three times lower. The axial load used in this study is based on the body weight of an average human being. In addition, the wear rate from this study was in the range of the wear rates (1.3–45.2 mm^3^/year) for retrievals investigated by Ellison and Fisher.^29^ However, an active comparison is difficult due to historic UHMWPE leading to failure mechanisms like delamination and oxidation, failure mechanisms which were prevalent in the retrievals study, but which are no longer common due to the use of the stabilised UHMWPE.

The wear scar areas were equal at the end of 9 MC for oval and 12 MC for round dome patellae buttons due to similar conformity generated between the buttons and femoral counterpart. The wear area measured for round dome (39.9% of the total articulating area) was larger than that estimated (30.7%) by earlier investigators.^24^ The lower wear scar area by Ellison et al.^24^ could have been due to a lower ML displacement, and potentially patellar tilt. However, the location and the shape of the wear scar from both the studies were similar. Schwartz et al.^44^ and Lindsey et al.^45^ suggested the ‘bow tie’ shape wear patterns in their retrieval studies. The same wear patterns were observed in this study (Figure 4) distributed along medial lateral quadrants.

With increasing number of cycles, the average roughness parameters of the patella buttons and femoral component increased. The femoral components became scratched parallel to the direction of FE rotation similar to studies of TKR.^19^ Wear characteristics like pitting, burnishing and scratching on the patella surface led to increase in roughness.

The wear volume was found to be proportional to the patellar tilt for every condition with rho value greater than 0.80. Patella tilt on the femoral counterpart likely caused higher cross shear, which may have led to higher volumetric wear.

This study has been able to identify the influence of the patella kinematics and design on the wear rate of the total knee joint replacement in a level gait cycle. More complexity in the kinematics will lead to understanding of complex phenomena like patella dislocation and loosening. The change in design (round or oval dome) did not result in a significant difference in wear rate at same kinematic conditions. The contribution of wear of the PFJ to overall TKR wear can lead to an increase in wear debris, which may lead to osteolysis and aseptic loosening of the joint.^17^ However, it should be emphasised that not only is the volumetric wear important but also size distribution of the particles and their biological activity.^18,30,42^ Ellison et al.^18^ found 90% of the wear debris in granular shape and size less than 1 µm similar to debris distribution in artificial tibia femoral joint.

This study was performed to investigate the effect of patellar rotation and ML displacements on the change in wear. With future musculoskeletal research in patella-femoral joint, further modification to the input kinematics can be proposed based on wide variation in in vivo kinematics.

## Conclusion

A six-axis in vitro simulator of the patella-femoral joint has been developed to investigate the wear of two designs under three kinematic conditions. Higher kinematic conditions through an increase in the AA rotation caused an increase in the wear rate in both designs of dome patellae buttons. However, an increase in ML displacement had less effect on the wear rates of both designs. The wear volume was positively correlated with patellar tilt with an increase in the patellar tilt, leading to an increase in the wear volume. This is the first preclinical wear simulation study test of PFJ in a six-axis simulator at varied kinematic conditions.
